# Antihyperglycemic Potential of *Grewia asiatica* Fruit Extract against Streptozotocin-Induced Hyperglycemia in Rats: Anti-Inflammatory and Antioxidant Mechanisms

**DOI:** 10.1155/2015/549743

**Published:** 2015-08-05

**Authors:** Hala A. H. Khattab, Nagla A. El-Shitany, Inas Z. A. Abdallah, Fatimah M. Yousef, Huda M. Alkreathy

**Affiliations:** ^1^Department of Food and Nutrition, Faculty of Home Economics, King Abdulaziz University, Jeddah, Saudi Arabia; ^2^Nutrition and Food Science Department, Faculty of Home Economics, Helwan University, Egypt; ^3^Department of Pharmacology and Toxicology, Faculty of Pharmacy, King Abdulaziz University, Jeddah, Saudi Arabia; ^4^Department of Pharmacology and Toxicology, Faculty of Pharmacy, Tanta University, Tanta, Egypt; ^5^Department of Pharmacology, Faculty of Medicine, King Abdulaziz University, Jeddah, Saudi Arabia

## Abstract

Diabetes mellitus is regarded as a serious chronic disease that carries a high risk for considerable complications. In folk medicine, the edible *Grewia asiatica* fruit is used in a number of pathological conditions. This study aimed to investigate the possible curative effect of *G. asiatica* fruit ethanolic extract against streptozotocin- (STZ-) induced hyperglycemia in rats. Furthermore, mechanism of antihyperglycemic action is investigated. Hyperglycemic rats are either treated with 100 or 200 mg/kg/day *G. asiatica* fruits extract. Serum glucose, liver glycogen, malondialdehyde (MDA), reduced glutathione (GSH), superoxide dismutase (SOD), interleukin- (IL-) 1*β*, and tumor necrosis factor- (TNF-) *α* are measured. *G. asiatica* fruits extract reduces blood glucose and pancreatic MDA levels. It increases liver glycogen and pancreatic GSH contents and SOD enzyme activity. Furthermore, *Grewia asiatica* fruits extract decreases serum IL-1*β* and TNF-*α*. The treatment also protects against STZ-induced pathological changes in the pancreas. The results of this study indicated that *G. asiatica* fruit extract exerts antihyperglycemic activity against STZ-induced hyperglycemia. The improvement in the pancreatic *β*-cells and antioxidant and anti-inflammatory effects of *G. asiatica* fruit extract may explain the antihyperglycemic effect.

## 1. Introduction

Diabetes mellitus (DM), as an important metabolic syndrome, has growing problem worldwide entailing enormous financial burden and medical care policy issues [1]. World Health Organization (WHO) has reported that there is 180 million diabetic in the world and that number will double by 2030 [2]. Because DM control without side effects is a challenge, drugs derived from plants may play an important role in the treatment of DM [3].

*Grewia asiatica* Linn. (*G. asiatica*) is commonly known as Phalsa or Falsa. It is a shrub or small tree with the fruit of 5–12 mm diameter, having purple to black color when it is ripe. It is cultivated primarily for its edible fruit and well reputed for its diverse medicinal uses. Edible fruit of* G. asiatica* in Indian folk medicine is used to alleviate blood disorders, inflammation, and cardiac and respiratory diseases [4]. It has been reported that* G. asiatica* possess anticancer [5], antioxidant [6], radioprotective [7], hepatoprotective [8], and antihyperglycemic [9] activities.* Grewia asiatica* fruit is a rich source of nutrients such as vitamins, minerals, and contain various bioactive compounds, like anthocyanins, tannins, phenolics, and flavonoids [10].

Inflammatory cytokines, such as interleukin-1* beta* (IL-1*β*) and tumor necrosis factor-alpha (TNF-*α*) secreted by the infiltrating immune cells, particularly CD8^+^ T cells, as well as reactive oxygen species (ROS) have been shown to play an important role in the pancreatic *β*-cell destruction and the insulitis that occurred in type 1 autoimmune diabetes [11]. Reactive oxygen species play an important role in the pathogenesis of diabetes, as sera from newly diagnosed type 1 DM patients exhibited increased reactivity of hydroxyl radical-modified glutamic acid decarboxylase 65 (GAD 65) [12]. Interestingly, recognition of GAD65 is even more pronounced with sera from patients suffering from the DM complications retinopathy and nephropathy, indicating that oxidation by ROS may generate a potent immunogenic molecule that drive type 1 diabetes [13].

Many studies have reported that plants rich in polyphenols, tannins, and flavonoids are effective scavengers of ROS [14, 15]. These natural products may prevent pancreatic *β*-cells destruction* via* inhibition of ROS production. Thus, it is a good strategy for the management of DM with plants that have antioxidant activities [16]. Therefore, this study aimed to evaluate first the antihyperglycemic effect of* G. asiatica* fruit ethanolic extract in STZ-induced diabetes in rats. Second, the possible antioxidant and immunomodulatory action of* G. asiatica* fruit ethanolic extract will be investigated.

## 2. Material and Methods

### 2.1. Plant Material

Fruits of* G. asiatica* are collected in August 2014 from Mazhar Botanic Garden, Baragil, Giza, Egypt. Fruits are identified by Professor Al-Nowaihi ASM, Faculty of Science, Botany Department, Ain Shams University, Cairo, Egypt.

### 2.2. Drugs and Chemicals

Streptozotocin (STZ) and chemicals with the highest laboratory purity are purchased from Sigma Chemical Co. (St. Louis, MO, USA). Kits are purchased from Sigma-Aldrich Co., USA.

### 2.3. Experimental Animals

Male Sprague Dawley rats (170–190 g) are obtained from the animal house of the National Research Center (NRC), Giza, Egypt. They are kept in standard laboratory conditions, fed on a standard AIN-93 diet [17], and given water* ad libitum*. They are handled in accordance with the standard guide for the care and use of laboratory animals in NRC.

### 2.4. Preparation of Ethanolic Extract of* G. asiatica* Fruit

Fruits of* G. asiatica* are washed and seeds are removed. The juicy pulp of each fruit is crushed and mixed in 95% ethanol. This procedure is repeated thrice. The crude ethanolic extract is filtered and evaporated under reduced pressure using a rotary evaporator [9]. It is stored in the refrigerator for further use.

### 2.5. Phytochemical Screening of* G. asiatica* Fruit Extract

The phytochemical analysis of* G. asiatica* fruit extract has been performed to find the presence of the major chemical constituents, including alkaloids, flavonoids, glycosides, saponins, tannins, resins, and triterpenoids using standard procedures of analysis [18].

### 2.6. Experimental Animals

Forty animals are divided into four groups (*n* = 8/group): (1) control: rats are i.p. injected with 0.2 mL of citrate buffer (0.05 M, pH 4.5); (2) hyperglycemia: rats are i.p. injected with STZ (65 mg/kg) [19]; (3) hyperglycemia +* G. asiatica* extract: hyperglycemia rats are treated orally (p.o.) with ethanolic extract of* G. asiatica* fruit (100 mg/kg/day) for 4 weeks; (4) hyperglycemia +* G. asiatica* extract: hyperglycemia rats are treated orally (p.o.) with ethanolic extract of* G. asiatica* fruit (200 mg/kg/day) for 4 weeks [9].

Rat's weight was detected at the beginning of the experiment and biweekly thereafter. The percent body weight gain (BWG %) was calculated. At the end of the experimental period, blood samples were collected from orbital plexus of vein, left to clot, and centrifuged at 3000 rpm for serum separations. Rats were euthanized using deep ether anaesthesia method; pancreas and liver tissues were dissected out.

### 2.7. Biochemical Analysis

Serum glucose [20], malondialdehyde (MDA) [21], reduced glutathione (GSH) [22], and superoxide dismutase (SOD) [23] were measured in homogenized pancreas. In addition, liver glycogen is determined [24].

### 2.8. Measurement of Cytokines TNF-*α* and IL-1*β*

Serum levels of TNF-*α* and IL-1*β* were measured by enzyme-linked immunosorbent assay (ELISA) using Assaypro TNF-*α* and IL-1*β* kits (30 Triad South Drive St. Charles, MO 63304, USA) using monoclonal antibodies specific for rat TNF-*α* and IL-1*β*, respectively. The primary antibody was biotin antibody and the assay Avidin D, Horseradish Peroxidase (Av-HRP) was used to bind the detection antibody, biotin with high affinity. Cytokine concentrations are calculated using a standard purified recombinant cytokines.

### 2.9. Histopathological Study

The formalin fixed pancreas are dehydrated in ascending grades of isopropyl alcohol and cleared in xylene. The sections are stained with Haematoxylin and Eosin (H&E) and examined microscopically.

### 2.10. Statistical Analysis

Results are reported as mean ± SDM. Data are subjected to one-way analysis of variance (ANOVA), followed by an L.S.D. post hoc multiple comparisons to determine the statistical significance of the difference using SPSS version 20.

## 3. Results

### 3.1. Phytochemical Screening of* G. asiatica* Fruit Extract

Phytochemical analysis of* G. asiatica* fruit extract reveals that it contains small amount of tannins and moderate amounts of alkaloids, saponins, and steroids. However, it contains large amounts of flavonoids, glycosides, and phenolic acids, whereas it is devoid of resins and triterpenoids (Table 1).

### 3.2. Effect of* G. asiatica* Fruit Extract on BWG% in Hyperglycemic Rats

Hyperglycemia causes a significant decrease (*p* < 0.001) in final body weight and BWG% compared to control group. Treatment of hyperglycemic rats with either 100 or 200 mg/kg* G. asiatica* fruit extract results in a significant (*p* < 0.001) increase in the final body weight and BWG% compared to hyperglycemic group in a dose-dependent manner. The high dose (200 mg/kg) induces biological functions modulation which is significantly different from the low dose (100 mg/kg) values. In addition, there are a nonsignificant difference between 200 mg/kg dose and the control group values, where it normalizes the biological evaluation parameters, Table 2.

### 3.3. Biochemical Analysis

The levels of blood glucose in control, hyperglycemic, and hyperglycemic treated with* G. asiatica* fruit extract are presented in Table 3. In the hyperglycemic group, a significant (*p* < 0.001) increase in blood glucose level is found compared to the control group. Oral administration of* G. asiatica* fruit extract (100 and 200 mg/kg) induces a significant (*p* < 0.001) decrease in blood glucose level compared to the hyperglycemic untreated group. The 200 mg/kg/day is more effective than the 100 mg/kg/dose in reducing blood glucose level.

The present study shows that liver glycogen level is significantly (*p* < 0.001) reduced in hyperglycemic group compared to the control group. Administration of* G. asiatica* fruit extract results in a significant (*p* < 0.001) increase in liver glycogen content compared to hyperglycemic group. The high dose (200 mg/kg) of* G. asiatica* fruit extract is more effective (*p* < 0.05) compared to the low dose (100 mg/kg) in normalizing the blood glucose level and liver glycogen content.

### 3.4. Antioxidant Status

The effect of* G. asiatica* fruit extract on lipid peroxidation detected as malondialdehyde (MDA), superoxide dismutase (SOD) enzyme activity, and reduced glutathione (GSH) in hyperglycemic rats is represented in Table 4. Hyperglycemia results in a significant (*p* < 0.001) elevation in pancreatic MDA and a significant (*p* < 0.001) decline in both pancreatic SOD and GSH compared to control group. On the other hand, oral administration with* G. asiatica* fruits extract to hyperglycemic rats at the two doses used improves the oxidative stress as manifested by the significant (*p* < 0.001) increase in SOD and GSH and the significant (*p* < 0.001) decrease in MDA compared to the hyperglycemic untreated group. The high dose (200 mg/kg) of* G. asiatica* fruit extract is more effective than the low dose (100 mg/kg), and there is a significant (*p* < 0.05) difference in MDA, GSH levels, and SOD activity between the two dosages used. Furthermore, high dose (200 mg/kg) normalizes the MDA, GSH, and SOD values, where there is a nonsignificant difference compared to the control group.

### 3.5. Effect of* G. asiatica* Fruits Extract on Cytokines IL-1*β* and TNF-*α* Concentration Measured in Hyperglycemic Rats

**Figure 1:**
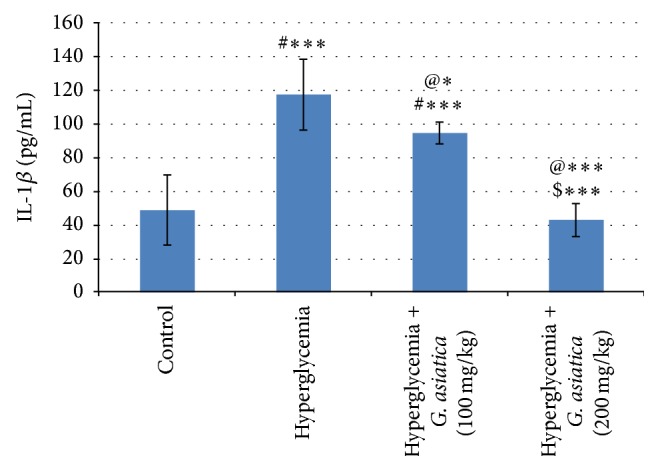
Effect of* G. asiatica* fruits extract on serum IL-1*β* concentration in hyperglycemic rats. Each value represents the mean of 8 rats ± SD. ^#^Significant difference between control and hyperglycemia group. ^@^Significant difference between hyperglycemia and hyperglycemia treated group. ^$^Significant difference between diabetics treated with 100 mg/kg and 200 mg/kg doses of* G. asiatica* fruit extract. ^*∗*^ *p* < 0.05, ^*∗∗*^ *p* < 0.01, and ^*∗∗∗*^ *p* < 0.001.

**Figure 2:**
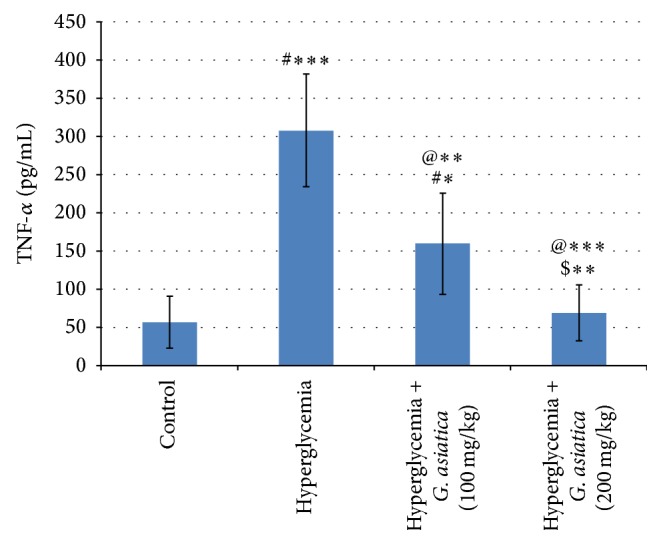
Effect of* G. asiatica* fruits extract on serum TNF-*α* concentration measured in hyperglycemic rats. Each value represents the mean of 8 rats ± SD. ^#^Significant difference between control and hyperglycemia groups. ^@^Significant difference between hyperglycemia and hyperglycemia treated groups. ^$^Significant difference between diabetics treated with 100 mg/kg and 200 mg/kg doses of* G. asiatica* fruit extract. ^*∗*^ *p* < 0.05, ^*∗∗*^ *p* < 0.01, and ^*∗∗∗*^ *p* < 0.001.

The present study shows that IL-1*β* and TNF-*α* serum levels are significantly (*p* < 0.001) increased in hyperglycemic untreated group compared to the control group. Administration of* G. asiatica* fruit extract in a dose of 100 mg/kg results in a significant decrease in serum IL-1*β* (*p* < 0.05) and TNF-*α* (*p* < 0.01) compared to hyperglycemic group. Administration of* G. asiatica* fruit extract in a dose of 200 mg/kg results in a significant (*p* < 0.001) decrease in serum IL-1*β* and TNF-*α* compared to hyperglycemic untreated group. The high dose (200 mg/kg) of* G. asiatica* fruit extract is more effective compared to the low dose (100 mg/kg) in normalizing the serum IL-1*β* (*p* < 0.001) and TNF-*α* (*p* < 0.01) (Figures 1 and 2).

### 3.6. Histopathological Examination

**Figure 3:**
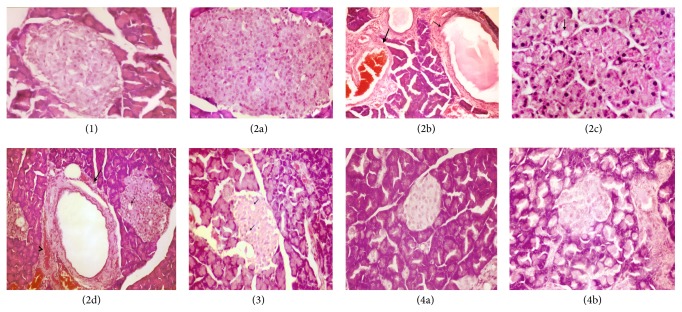
Pancreas of control group (1) showing no histopathological changes. Sections ((2a)–(2d)): pancreas of hyperglycemic rats showing *β*-cells hyperplasia of Langerhans islets (2a), dilatation of pancreatic duct (small arrow) and congestion of blood vessel (large arrow) (b), vacuolar degeneration of epithelial lining pancreatic acini associated with pyknosis of their nuclei (2c), and vacuolation of sporadic of *β*-cells of Langerhans islets (small arrow), cystic dilatation of pancreatic duct (large arrow), and congestion of periductal blood vessel (arrow head) (2d). Section (3): pancreas of hyperglycemic +* G. asiatica* fruit extract (100 mg/kg) group showing vacuolation *β*-cells of Langerhans islets. Sections ((4a) and (4b)): pancreas of hyperglycemic +* G. asiatica* fruit extract (200 mg/kg) group showing no apparent histopathological changes (H&E ×400).

Microscopical examination of the pancreas of control group reveals a normal histopathological structure of pancreas cells (Figure 3(1)). Meanwhile, pancreas of hyperglycemic group shows hyperplasia of *β*-cells of islets of Langerhans (Figure 3(2a)), dilatation of pancreatic duct and congestion of blood vessel (Figure 3(2b)), vacuolar degeneration of epithelial lining pancreatic acini associated with the pinkness of their nuclei (Figure 3(2c)), and vacillation of sporadic of *β*-cells of islets of Langerhans, cystic dilatation of pancreatic duct, and congestion of periductal blood vessel (Figure 3(2d)). Pancreas of hyperglycemic rats treated with 100 mg/kg* G. asiatica* fruit extract reveals vacuolation *β*-cells of islets of Langerhans (Figure 3(3)). While pancreas cells of hyperglycemic treated with 200 mg/kg* G. asiatica* fruit extract reveal improvement in histopathological findings (Figures 3(4a) and 3(4b)).

## 4. Discussion

Diabetes mellitus encompasses a heterogeneous group of disorders characterized by insulin hyposecretion and/or insensitivity [25]. In spite of the fact that insulin is the most important therapeutic agent known to medicine, researchers have been making efforts to find insulin-like substances from plant sources for the treatment of diabetes [26].

The antioxidants present in fruits show strong activity against cancer, cardiovascular, and various chronic diseases.* Grewia asiatica* is an important medicinal plant and its fruits have been used in the treatment of various diseases. Phytochemical screening of* G. asiatica* fruit extract reveals that it contains flavonoids, glycosides, saponins, and phenolic acids. The present results are in line with those of Zia-Ul-Haq et al., who have reported that* G. asiatica* fruit extract has many bioactive components as glycosides, saponins, and steroids [10]. Moreover, methanol extract of* G. asiatica* antioxidant activity is evaluated by various assays. The results indicated that fruit possesses considerable antioxidant activities and contains high amounts of total flavonoid, phenolic, and anthocyanin [27].

Diabetes induced reduction in body weight. The body's inability to store or use glucose causes hunger and weight loss [28]. In our study, administration of* G. asiatica* fruit extract in the two treated doses increases the body weight, indicating its beneficial effect in preventing body weight loss in diabetic rats [29]. The effect of* G. Asiatica* fruit extract in preventing body weight loss seems to be attributed to its ability to reduce hyperglycemia, which may be due to its antioxidant and radical scavenging activity [7].

The obtained results reveal that oral administration of* G. asiatica* fruit extract to hyperglycemic rats decreased both blood glucose and liver glycogen. The antihyperglycemic effect of* G. asiatica* fruit extract has been reported by Parveen et al.; the effect of* G. asiatica* fruit extract may be due to its bioactive antioxidant constituents as shown by phytochemical analysis [9]. It has been reported that many bioactive principles possess antihyperglycemic activity as alkaloids, saponins, tannins, and phenolics [30]. Diabetes management can be achieved by delaying enzyme *α*-amylase activity [31]. Gupta et al. [5] have reported that methanolic extracts of* G*.* asiatica* fruit residues show *α*-amylase inhibitory activity. Das et al. [32] show that *α*-amylase inhibition property can be achieved by flavonoid.

Increasing ROS production plays an important role in the development and progression of hyperglycemia [33]. Our results show that hyperglycemia increases pancreatic MDA and decreases GSH and SOD activities. These effects might be due to the fact that hyperglycemia increases oxidative stress through ROS overproduction [34]. Treatment of hyperglycemic rats with* G. asiatica* fruit extract improves pancreas tissue contents of MDA, GSH, and SOD. Asghar et al. [6] also found that* G. asiatica* fruit has a potent antioxidant activity* in vitro*. Results of Sisodia et al. [35] have indicated that* G. asiatica* fruit pulp extract protects mice blood against radiation-induced damage. In addition, Sharma and Sisodia have reported that* G. asiatica* fruit extract contains anthocyanin and bioactive compounds that are found to have strong radical scavenging activity in 2,2-diphenyl-1-picrylhydrazyl (DPPH) and O_2_^−^ assays [7]. It shows also* in vitro* radioprotective activity in protein carbonyl assay in a dose-dependent manner. Streptozotocin generated ROS that contribute to DNA fragmentation and evoke other deleterious changes in *β*-cells [36].

There is increasing evidence that a strong relationship has been found between the inflammatory processes, the subsequent *β*-cell dysfunction, and insulin signaling impairment [37]. TNF-*α* is a pleotropic peptide that plays an important role in several inflammatory and cytotoxic reactions [38]. Our results show that* G. asiatica* fruit extract decreases IL-1*β* and TNF-*α* induced during hyperglycemia. Paviaya et al. [39] have reported that* G. asiatica* shows an anti-inflammatory effect in carrageenan-induced rat hind paw edema model. In line with our results, quercetin suppresses the inflammatory process in aortic tissues in two different diabetic models via amelioration of the elevation in TNF-*α* [40]. The flavonoid quercetin is one of the major constituents of* G. asiatica* fruit [41].

## 5. Conclusion

In conclusion, the present study demonstrates that ethanolic extract of* G. asiatica* fruit possesses significant hypoglycemic, antioxidant, and immunomodulatory effects. The presence of several bioactive compounds in this plant extract, particularly flavonoids and phenolic acids, might be responsible for these effects. Therefore, the extract should be further investigated as a new supplement for the management of diabetes with minimal side effects.

## Figures and Tables

**Table 1:** Phytochemical screening of *G. asiatica* fruit extract.

Phytochemical tests	Results
Alkaloids	++
Flavonoids	+++
Glycosides	+++
Steroids	++
Saponins	++
Tannins	+
Phenolic acids	+++
Resins	−
Terpenoids	−

The following symbol indicated the intensity of active compounds: absence of the constituents (−), small amount (+), moderate amount (++), and large amount (+++).

**Table 2:** Effect of* G. asiatica *fruits extract on biological evaluation in hyperglycemic rats.

Experimental groups	Control	Hyperglycemic	Hyperglycemic + *G. asiatica* (100 mg/kg)	Hyperglycemic + *G. asiatica* (200 mg/kg)
Initial BW (g)	179.41 ± 4.40	178.87 ± 4.05	180.20 ± 3.88	179.27 ± 5.98
Finial BW (g)	262.65 ± 4.37	199.16 ± 5.82^a*∗∗∗*^	251.35 ± 7.51^a*∗∗*b*∗∗∗*^	258.49 ± 6.72^b*∗∗∗*c*∗*^
BWG%	46.40 ± 4.17	11.34 ± 1.14^a*∗∗∗*^	39.48 ± 3.83^a*∗∗*b*∗∗∗*^	44.19 ± 4.41^b*∗∗∗*c*∗*^

Results represent mean of 8 rats ± SED.

^a^Significant difference between control and hyperglycemic groups.

^b^Significant difference between hyperglycemic and hyperglycemic treated groups.

^c^Significant difference between hyperglycemic treated with 100 mg/kg and 200 mg/kg of *G. asiatica *fruit extract.

(^*∗*^ *p* < 0.05, ^*∗∗*^ *p* < 0.01, and ^*∗∗∗*^ *p* < 0.001).

**Table 3:** Effect of* G. asiatica *fruits extract on serum glucose and liver glycogen in hyperglycemic rats.

Experimental groups	Control	Hyperglycemic	Hyperglycemic + *G. asiatica* (100 mg/kg)	Hyperglycemic + *G. asiatica* (200 mg/kg)
Glucose (mg/dL)	99.66 ± 8.25	150.59 ± 10.96^a*∗∗∗*^	116.74 ± 10.81^a*∗∗*b*∗∗∗*^	105.15 ± 10.47^b*∗∗∗*c*∗*^
Liver glycogen (mg/g tissue)	8.65 ± 0.53	6.52 ± 0.51^a*∗∗∗*^	7.66 ± 0.65^a*∗∗*b*∗∗∗*^	8.37 ± 0.59^b*∗∗∗*c*∗*^

Results represent mean of 8 rats ± SED.

^a^Significant difference between control and hyperglycemic groups.

^b^Significant difference between hyperglycemic and hyperglycemic treated groups.

^c^Significant difference between hyperglycemic treated with 100 mg/kg and 200 mg/kg of *G. asiatica* fruit extract.

(^*∗*^ *p* < 0.05, ^*∗∗*^ *p* < 0.01, and ^*∗∗∗*^ *p* < 0.001).

**Table 4:** Effect of* G. asiatica *fruits extract on lipid peroxidation (MDA), reduced glutathione (GSH), and superoxide dismutase (SOD) enzyme activities in hyperglycemic rats.

Experimental groups	Control	Hyperglycemic	Hyperglycemic + *G. asiatica* (100 mg/kg)	Hyperglycemic + *G. asiatica* (200 mg/kg)
MDA (*μ*mol/g tissue)	71.62 ± 5.95	113.89 ± 8.73^a*∗∗∗*^	81.14 ± 6.61^a*∗*b*∗∗∗*^	75.50 ± 6.31^b*∗∗∗*^
GSH (mg/g tissue)	56.18 ± 5.59	33.88 ± 3.37^a*∗∗∗*^	49.76 ± 4.77^a*∗*b*∗∗∗*^	53.33 ± 4.97^b*∗∗∗*^
SOD (U/g tissue)	66.25 ± 4.89	37.50 ± 3.47^a*∗∗∗*^	59.89 ± 5.20^a*∗*b*∗∗∗*^	63.94 ± 4.77^b*∗∗∗*^

Results represent mean of 8 rats ± SED.

^a^Significant difference between control and hyperglycemic groups.

^b^Significant difference between hyperglycemic and hyperglycemic treated groups.

(^*∗*^ *p* < 0.05, ^*∗∗*^ *p* < 0.01, and ^*∗∗∗*^ *p* < 0.001).
